# Intermittent fasting, energy balance and associated health outcomes in adults: study protocol for a randomised controlled trial

**DOI:** 10.1186/s13063-018-2451-8

**Published:** 2018-02-02

**Authors:** Iain Templeman, Dylan Thompson, Javier Gonzalez, Jean-Philippe Walhin, Sue Reeves, Peter J. Rogers, Jeffrey M. Brunstrom, Leonidas G. Karagounis, Kostas Tsintzas, James A. Betts

**Affiliations:** 1 Department for Health, University of Bath, Bath, BA2 7AY UK; 2 Department of Life Sciences, University of Roehampton, London, SW15 4JD UK; 3 School of Experimental Psychology, University of Bristol, Bristol, BS8 1TU UK; 4 Faculty of Sport and Health Sciences, University of St Mark and St John, Plymouth, PL6 8BH UK; 5 School of Life Sciences, University of Nottingham, Queen’s Medical Centre, Nottingham, NG7 2UH UK

**Keywords:** Intermittent fasting, Calorie restriction, Energy balance, Obesity, Metabolism, Physical activity, Body composition, Postprandial

## Abstract

**Background:**

Prior studies have shown that intermittent fasting is capable of producing improvements in body weight and fasted health markers. However, the extent to which intermittent fasting incurs compensatory changes in the components of energy balance and its impact on postprandial metabolism are yet to be ascertained.

**Methods:**

A total of 30–36 lean participants and 30–36 overweight/obese participants will be recruited to provide two separate study groups who will undergo the same protocol. Following an initial assessment of basic anthropometry and key health markers, measurements of habitual energy intake (weighed food and fluid intake) and physical activity energy expenditure (combined heart rate and accelerometry) will be obtained over 4 weeks under conditions of energy balance. Participants will then be randomly allocated to one of three experimental conditions for 20 days, namely (1) daily calorie restriction (reduce habitual daily energy intake by 25%), (2) intermittent fasting with calorie restriction (alternate between 24-hour periods of fasting and feeding to 150% of habitual daily energy intake), (3) intermittent fasting without calorie restriction (alternate between 24-hour periods of fasting and feeding to 200% of habitual daily energy intake). In addition to continued monitoring of energy intake and physical activity during the intervention, participants will report for laboratory-based assessments of various metabolic parameters both before and after the intervention. Specifically, fasting and postprandial measurements of resting metabolic rate, substrate oxidation, appetite, food preference, and plasma concentrations of key metabolites and hormones will be made, in addition to subcutaneous abdominal adipose tissue biopsies in the fasted state and an assessment of body composition via dual-energy x-ray absorptiometry.

**Discussion:**

Comparing observed changes in these measures across the three intervention arms in each group will establish the impact of intermittent fasting on postprandial metabolism and the components of energy balance in both lean and overweight/obese populations. Furthermore, this will be benchmarked against current nutritional interventions for weight management and the relative contributions of negative energy balance and fasting-dependent mechanisms in inducing any observed effects will be elucidated.

**Trial registration:**

Trial retrospectively registered at clinicaltrials.gov under reference number NCT02498002 (version: IMF-02, date: July 6, 2015).

**Electronic supplementary material:**

The online version of this article (10.1186/s13063-018-2451-8) contains supplementary material, which is available to authorized users.

## Background

Intermittent fasting (IMF) is a dietary strategy in which periods of normal food and drink consumption are punctuated by periods of energy restriction or fasting. The objective of IMF is to create a net reduction in energy intake that causes it to fall below energy expenditure, thereby creating a state of negative energy balance and inducing weight loss [1]. Work by various groups has consistently shown that such an approach is associated with significant reductions in body mass in obese participants, whilst also improving blood lipid profile and lowering concentrations of inflammatory markers [2–4]. Although these findings are broadly comparable to those seen following a period of daily calorie restriction [5–13], current understanding of how IMF affects human health and metabolism is far from complete.

Firstly, while the weight losses reported in prior studies of IMF reflect a state of negative energy balance, the precise changes that occur in the components of energy balance have not been characterised. This is of importance as prolonged periods of daily calorie restriction, which also aim to create a state of negative energy balance, elicit compensatory reductions in energy expenditure and increases in appetite that preserve body fat stores and undermine weight loss efforts [14–22]. Although this adaptive phenomenon has been thoroughly documented in studies of daily calorie restriction, there is no evidence to suggest that such a mechanism is invoked in response to IMF. Changes in leptin concentration, a proposed hormonal regulator of energy balance [23], have been shown to be similar between IMF and daily calorie restriction [2, 4, 11, 24]. However, this does not seem to translate to measurable changes within the components of energy balance, with studies failing to detect corresponding reductions in resting metabolic rate [25] or habitual physical activity [26]. Although promising, these prior studies have focused on isolated aspects of energy expenditure that do not accommodate the complex interplay between the components of energy balance [1, 21], thus warranting closer examination.

Secondly, reductions in fasting insulin concentration have been consistently shown in response to various IMF interventions as reviewed by Barnosky et al. [8]. This review also highlights that, in a subset of these studies, fasted indices of insulin resistance generally improve following a period of IMF. However, it is important to note that these fasted measurements can often overlook subtle modifications in glucose metabolism under dynamic conditions [27] such as those seen in the postprandial period. At present there is a dearth of knowledge on how IMF impacts postprandial metabolism which needs to be addressed, especially upon considering that Western cultures spend the majority of their waking time in a postprandial state [28]. Furthermore, discerning the relative contributions of fasting and negative energy balance to any observed changes in metabolism carries substantial implications for how obesity and the associated comorbidities are treated, as it has been suggested that temporal modifications in energy intake exert independent influences on metabolic health [29–32].

With over 50% of the adult population in the UK currently classified as overweight or obese [33], accompanied by an increased risk of type 2 diabetes and cardiovascular disease, finding more effective strategies to manage these conditions remains imperative [34]. Although the limited body of prior literature offers promising insights as to the efficacy of IMF in improving metabolic health, numerous uncertainties remain. As such, the present study has three core objectives that will be separately addressed in both a lean cohort and in an overweight/obese cohort.

The study will aim (1) to establish whether IMF elicits compensatory changes in the components of energy balance and compare these against the changes arising from a daily calorie restriction intervention; (2) to examine the effect of IMF on postprandial metabolism relative to a daily calorie restriction intervention; and (3) to explore whether IMF impacts upon postprandial metabolism and the components of energy balance independently from chronic energy imbalance by contrasting against a eucaloric IMF intervention.

## Methods

### Participants

To address these objectives two separate cohorts of 30–36 healthy adults who satisfy the inclusion/exclusion criteria outlined below will be recruited. One cohort will feature exclusively lean participants (body mass index (BMI) 20.5–25.0 kg/m^2^) and the other overweight/obese participants (BMI > 25.0 kg/m^2^). This will be confirmed using the fat mass index (FMI) obtained from a dual-energy x-ray absorptiometry (DEXA) scan, with values ≤ 7.5 kg/m^2^ and ≤ 11.0 kg/m^2^ classified as lean for males and females, respectively. In line with the objectives of this protocol the effects of IMF in these two cohorts will not be compared, instead they will be published as separate studies with matched methods to provide a thorough evaluation of the impacts of IMF in these two metabolically distinct populations.

Participants in each cohort shall then be allocated to one of three parallel intervention arms in a 1:1:1 allocation ratio using a stratified randomisation scheme. This will feature a factor for objectively measured physical activity level (PAL), which is calculated by dividing each participant’s total daily energy expenditure by their resting metabolic rate, to ensure an even distribution of lower activity (PAL < 1.75) and higher activity (PAL ≥ 1.75) participants in each condition. In accordance with best practice recommendations for randomised controlled trials [35], allocation concealment will be employed to minimise bias. The scheme will be generated by a senior author who is not involved in participant recruitment or management (JPW) using an electronic random number generator. Intervention allocation will be requested via email by those responsible for participant management (IT), with only the participant code, BMI/FMI and PAL being sent. As only the delivery team will engage with participants and know their corresponding code, whilst only the senior author knows the block sizes and sequences, it is not possible for the allocation to be reliably predicted or biased by either party. Full details of the overall randomisation scheme will be included in publications once all allocations are completed in order to prevent deciphering [36]. The protocol for these studies has received ethics approval from the National Health Service Research Ethics Committee (reference: 15/SW/0007) and has been registered at clinicaltrials.gov under reference number NCT02498002 (version: IMF-02, date: July 6, 2015).

#### Inclusion

Participants (1) aged 18–65 years, (2) with a BMI of 20.5–25.0 kg/m^2^ (lean) or > 25.0 kg/m^2^ (overweight/obese), (3) a stable body weight (±3 kg) for at least 6 months [37], (4) able and willing to comply with study procedures, (5) willing to undertake required fasting durations, and (6) with the capacity to provide informed consent will be included in the study.

#### Exclusion

Participants (1) with body weight > 120 kg, (2) recent or planned engagement in fasting practices (within 3 months of start date), (3) recent or planned change in diet/physical activity habits, (4) suffering from an eating disorder as assessed using the Eating Disorder Examination Questionnaire (EDE-Q 6.0) [38], (5) diagnosed with diabetes or other metabolic health disturbances, (6) with an ongoing medical condition or treatment that may interfere with study variables, (7) menopausal, (8) pregnant, recently pregnant, planning to become pregnant (within 3 months) or currently breastfeeding, (9) having donated blood within last 3 months, (10) with a lack of capacity/language skills to independently follow the protocol, (11) who cannot consume test meals due to intolerances/dietary preferences (i.e. vegan, gluten, milk proteins), or (12) with any other behaviour or condition that may introduce bias to the study or poses undue personal risk, will be excluded from the study.

### Power calculation

The sample size for this study has been estimated using an a priori power analysis of studies employing similar durations of daily calorie restriction. Specifically, Friedlander et al. [39] observed a significant decline in resting metabolic rate following 21 days of daily calorie restriction (pre: 1898 ± 262 kcal/day, post: 1670 ± 203 kcal/day). Applying a two-tailed *t* test to these data suggests that 11 participants would be required to achieve 80% power when detecting such an effect at an alpha level of 0.05. Focusing instead on changes in postprandial glucose metabolism, Molfino et al. [40] reported a significant reduction in 2-hour post-bolus plasma glucose values during an oral glucose tolerance test following 10 days of daily calorie restriction (pre: 10.72 ± 3.56 mmol/L, post: 7.10 ± 2.96 mmol/L). In this instance, nine participants would be required to detect a comparable within-treatment effect with 80% power at an alpha level of 0.05. Collectively, this suggests that 9–11 participants will be required per group to establish within-treatment effects in the primary outcomes. However, given the potential for variability in the outcomes of interest, particularly when working with obese populations [16, 17], a more conservative requirement of 10–12 was decided upon. With 10–12 participants required in each of the three intervention arms, 30–36 lean participants and 30–36 overweight/obese participants will be sought in total, allowing examination of these two metabolically distinct populations to be kept separate. Recruitment will be conducted using adverts at local employers and media outlets, and will proceed on a rolling basis until an adequate sample size is reached. To minimise loss to follow-up emphasis will be placed on considering the demands of the study before enrolling and that feedback of personal results will only be available to those who complete all three lab sessions. In cases of withdrawal, additional participants will be sought to maintain statistical power; however, the point of withdrawal and the accompanying reason will be reported in a CONSORT flow diagram in subsequent publications.

### Experimental protocol

#### Overview

**Fig. 1:**
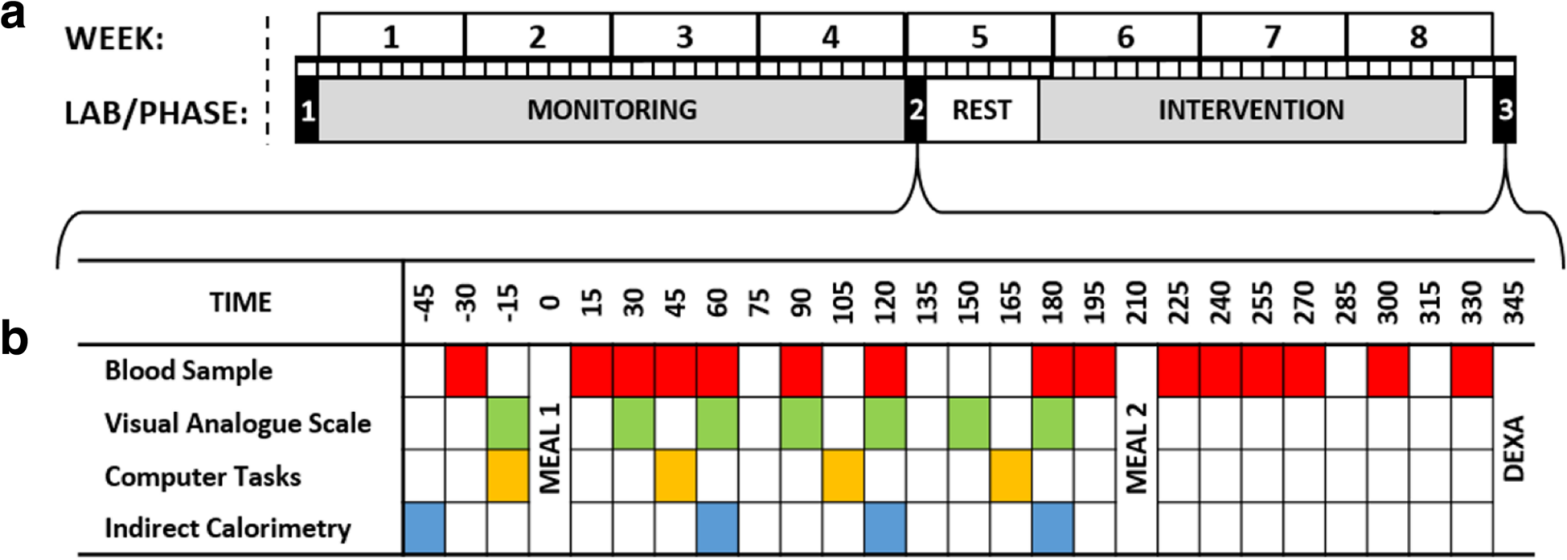
Schematic of the 8-week study design (**a**) and the sampling intervals for laboratory sessions 2 and 3 (**b**). *DEXA* dual-energy x*-*ray absorptiometry

**Fig. 2:**
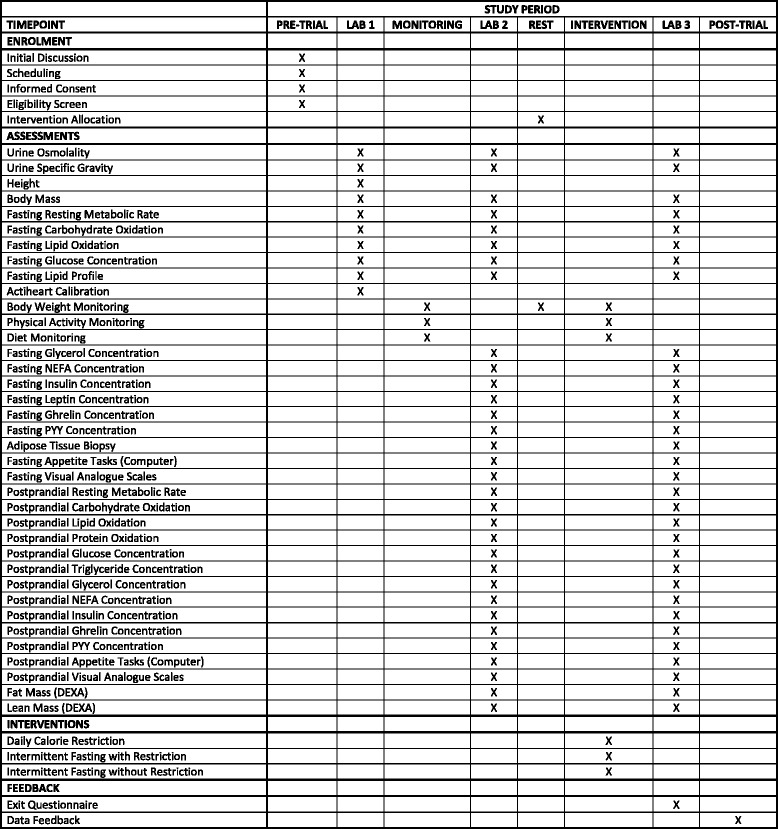
SPIRIT 2013 figure showing schedule of enrolment, assessments, allocation and intervention for each participant. *NEFA* non-esterified fatty acids, *PYY* peptide YY, *DEXA* dual-energy x-ray absorptiometry

Having read the participant information sheet (Additional file 1) and discussed the study in its entirety with a member of the research team (IT), potential participants will be guided through the process for providing written informed consent (Additional file 2). Following this, eligibility will be assessed by a series of self-report questionnaires together with a BMI calculation. Eligible participants will then undergo the 8-week protocol shown in Fig. 1a. All lab sessions will be completed at the University of Bath (Somerset, UK) whilst all other aspects of the study will occur under free-living conditions. In preparation for all laboratory sessions, participants will avoid caffeine, alcohol, smoking and strenuous exercise throughout the preceding 24 hours, whilst also standardising their dietary intake. Following an overnight fast (minimum 10 hours) participants will report to the laboratory at 07:30 ± 01:00 having consumed 500 mL of water upon waking. Female participants will schedule all laboratory sessions to coincide with the follicular phase of their menstrual cycle (i.e. 3–10 days after onset of menses) to account for monthly fluctuations in hormone levels and the associated changes. A SPIRIT figure showing the schedule of enrolment, assessment of outcome measures, allocation and interventions across the 8-week timeline is shown in Fig. 2, and the accompanying SPIRIT checklist can be found in Additional file 3.

#### LAB 1 (baseline)

This initial lab session will provide a reference point for examining the stability of body mass, as an indicator of overall energy balance, throughout the ensuing 4-week monitoring phase in which habitual dietary intake and physical activity are quantified. In addition, this visit will serve to familiarise participants with key procedures to improve reliability over subsequent laboratory sessions [41, 42]. Specifically, this will involve the provision of a urine sample to measure hydration status before both height and body mass are measured. Resting metabolic rate and substrate oxidation will be quantified using indirect calorimetry of expired air samples, after which a fasted blood sample shall be drawn from the antecubital vein to measure baseline concentrations of certain biomarkers (e.g. glucose, cholesterol). To conclude this session, a supervised submaximal treadmill protocol will also be undertaken to individually calibrate the physical activity monitors being used throughout the study (Actiheart™).

#### Monitoring phase

During this 4-week phase, participants will be asked to maintain their habitual diet and activity patterns. Within this, both energy intake and physical activity will be measured concurrently in four designated monitoring windows of 3 consecutive days each. These windows will be separated by at least 2 days and the final window will coincide with the 3 days leading up to LAB 2. Data on physical activity energy expenditure and intensity will be captured using individually calibrated Actiheart^TM^ monitors [43, 44], whilst energy intake will be measured by means of a weighed record of food and fluid intake. To proceed into the next phase, participants will be required to maintain energy balance throughout the monitoring phase and provide accompanying measurements of energy intake and expenditure. Energy balance will be ascertained by maintaining a stable body weight (<1.0 kg increase or decrease) between LAB 1 and LAB 2 [16, 45].

#### LAB 2 (pre-intervention)

Having completed the monitoring phase, participants will then return to the laboratory for measurement of a series of fasted and postprandial outcomes. Once again, hydration status will be ascertained from a urine sample before body mass is measured. As discussed previously, if body mass differs from the value recorded in LAB 1 by more than 1.0 kg then participants will be asked to repeat the monitoring phase. Providing this criterion is satisfied, then fasting measurements of resting metabolic rate and substrate oxidation will be repeated before a cannula is fitted to an antecubital vein and a fasting venous blood sample is drawn. In order to examine changes at the tissue level, a subcutaneous adipose tissue biopsy will also be obtained from the abdominal region at this time. The last of these fasted measurements will be a set a visual analogue scales and computer-based tasks to assess perceived hunger, fullness, desire to eat and food preferences. This will then be followed by two sequential test meals. The first will feature indirect calorimetry measurements from expired gases, visual analogue scales/computer tasks, and venous blood samples, whereas the second will only involve sampling of arterialised venous blood. Figure 1b shows the sampling intervals for the various measurements. Blood samples will be used to examine both postprandial nutrient handling (e.g. glucose, insulin, triglyceride concentrations) and appetite regulation (e.g. ghrelin, peptide YY concentrations). To conclude, a DEXA scan will be performed to measure pre-intervention fat and lean tissue mass.

#### Intervention phase

During a 6-day rest following LAB 2, which is included to avoid prolonged periods of monitoring [46] and maintain the 4-week testing interval, participants will be randomised to one of the three parallel intervention arms outlined in Table 1. The three diet groups will last for 20 days each with transitions between 24-hour cycles occurring at 15:00 each day. Not only does this transition point allow the consumption of at least one main meal per day to aid motivation, but more importantly, it also ensures that the latter and more demanding part of the fast coincides with diurnal reductions in appetite to encourage adherence [47]. Furthermore, this structure still gives a full 24-hour fasting period, which is around double that achieved in most prior studies [8].

**Table 1:** Intervention arms employed in the study protocol

Intervention	Description
Daily calorie restriction	Reduce normal intake by 25% every day
Intermittent fasting with calorie restriction	Alternate between 24-hour periods of fasting and feeding with 150% of normal intake on fed days
Intermittent fasting without calorie restriction	Alternate between 24-hour periods of fasting and feeding with 200% of normal intake on fed days

For the IMF groups, during fasted cycles, participants are only permitted water, herbal teas and black tea/coffee with no sugar (i.e. unsweetened energy-free drinks). During feeding cycles, and throughout the daily calorie restriction diet, participants will appropriately modify their normal diet to give 75%, 150% or 200% of their habitual daily energy intake (measured during monitoring phase) in accordance with the group they are assigned to. Participants will also continue to monitor their physical activity and energy intake over the first and last 6 days of the intervention period. In the context of physical activity, this is to examine behavioural adaptations to the different diets; therefore, participants are allowed to adjust the quantity of activity/exercise undertaken based on how they feel, but are instructed not to incorporate any unfamiliar activities. In contrast, measurements of energy intake will be used to monitor compliance with intake targets with feedback provided to maximise accuracy. It is anticipated that, for some participants, doubling the volume of their habitual diet to achieve the calorie target whilst maintaining macronutrient balance will not be feasible. In these circumstances, achieving the required calorie intake will take precedence over maintaining macronutrient balance, both of which will be reported in resulting publications.

#### LAB 3 (post-intervention)

Following the completion of 20 consecutive 24-hour dietary cycles, participants will be required to standardise their diet and activity levels for 1 day to match those reported in the lead up to previous lab sessions and eliminate any residual effects of the final diet period. As with LAB 1 and LAB 2, this will also involve avoiding caffeine, alcohol, smoking and strenuous exercise before completing an overnight fast (minimum 10 hours) and consuming 500 mL of water upon waking. At 07:30 ± 01:00, participants will return to the laboratory and repeat the protocol outlined earlier for LAB 2, thereby providing a pre–post comparison.

### Anthropometry

Having voided upon arrival, measurements of body mass will be taken to the nearest 0.05 kg (Weylux 424, UK) with participants wearing light clothing. Height will be measured using a wall-mounted stadiometer to the nearest 0.1 cm whilst participants stand with their heels and shoulders touching the stadiometer. Body composition will be assessed by means of a DEXA scan (Discovery W, Hologic, MA, USA) taken in a supine position. For this, participants will be asked to lie on the scanning table with their feet evenly spaced to either side of the midline. Arms will be placed by their sides and equidistant to the trunk with the hands pronated and fingers spread. All measurements will be made following a quality control scan of a spine phantom with known properties in accordance with the manufacturer’s instructions.

### Energy intake

Energy intake will be measured throughout both the monitoring and intervention phases by means of a weighed record of food and fluid intake. In the initial lab session, participants shall be provided with a set of compact kitchen scales (SmartWeigh^TM^ Pocket Pro, NY, USA) and a log book to record all food and drink items. In addition, a researcher will discuss best practice with them and emphasise the level of detail required. These weighed records will be analysed (Nutritics^TM^, Ireland) to yield values for energy and macronutrient intake with all records for a given participant analysed by the same researcher.

### Physical activity

Physical activity will be measured using Actiheart™ monitors (CamNtech, UK) that employ combined heart rate and accelerometry data to yield minute-by-minute estimates of physical activity energy expenditure and intensity under free-living conditions [43]. These monitors will be individually calibrated for each participant using an adapted version of the treadmill protocol described by Brage et al. [44]. Specifically, this involves completing four 3-minute stages of supervised incremental treadmill locomotion, namely level walking at 3.2 km/h, brisk level walking at 5.2 km/h, brisk graded walking at 5.8 km/h (+10%) and level running at a self-selected speed of 9.0–12.0 km/h. During the final minute of each stage, heart rate (RS400, Polar, Finland) and energy expenditure (indirect calorimetry of expired air samples) will be measured. This will yield a heart rate–physical activity intensity regression equation for each participant which accommodates the inter-individual differences in this relationship to dramatically improve concurrent validity [44]. However, participants can choose to omit the final stage (level running) if they do not feel able in order to avoid biasing the sample in favour of more formal exercisers. Furthermore, the treadmill protocol will also be ended prematurely if the participant asks to stop or if their heart rate exceeds 85% of their age-predicted maximum.

### Meal tests

In order to examine the impact of the interventions on nutrient handling and appetite regulation, in addition to any second meal effects [48], two successive meal tests will be completed in LAB 2 and LAB 3. Both meals will be tailored to provide one-third of resting metabolic rate as measured in the fasted state during LAB 2. The first meal (breakfast) will be in the form of a homogenous porridge meal (1.31 kcal/g; 59% carbohydrate, 29% fat, 12% protein) composed of golden syrup flavour instant oats (Sainsbury’s, UK), whole milk (Tesco, UK) and white granulated sugar (Silver Spoon, UK). This will be cooked in a microwave and cooled for 10 minutes before being consumed in its entirety within a designated 10-minute eating opportunity. This will be followed by a 3-hour postprandial period featuring venous blood samples (15, 30, 45, 60, 90, 120, 180 minutes), visual analogue scales (30, 60, 90, 120, 150, 180 minutes), computer-based food-choice tasks (45, 105, 165 minutes) and expired air samples (60, 120, 180 minutes).

By comparison, the second meal (lunch) will take the form of a liquid meal-replacement supplement (1.50 kcal/mL; 54% carbohydrate, 30% fat, 16% protein; Ensure Plus, Abbott Nutrition, OH, USA) which will be consumed following a blood sample within a 5-minute feeding window positioned 210 minutes after the consumption of the first meal. A 2-hour postprandial period will then follow in which arterialised venous blood samples will be drawn using the heated hand-box method (225, 240, 255, 270, 300, 330 minutes).

### Indirect calorimetry

Resting metabolic rate and substrate oxidation in both the fasted and postprandial state will be measured using indirect calorimetry of expired air samples [49]. In each instance, three consecutive 5-minute air samples will be collected using a mouthpiece and the Douglas bag method (Hans Rudolph, MO, USA) before analysis (Servoflex MiniHF 5200, Servomex, UK) and evacuation (Dry Gas Meter, Harvard Apparatus, MA, USA). Samples will be collected in accordance with best practice guidelines [50], with the values from two or more samples that agree to within 100 kcal/day averaged and used in further analysis [37], along with the corresponding substrate oxidation parameters. During the treadmill test for the calibration of activity monitors, a single 1-minute air sample will be collected per stage for which a facemask shall be used in place of the mouthpiece for participant comfort.

### Blood sampling

In LAB 2 and LAB 3, all blood samples will be procured by means of an intravenous cannula. At each sampling interval, the first 2 mL of the draw will be discarded as waste before a 5 mL sample is collected. This sample will then be dispensed in to an EDTA tube and centrifuged before the supernatant is aliquoted across three 1.5 mL tubes and stored at −80 °C pending subsequent analysis for plasma concentrations of nutrients (e.g. glucose, triglycerides) and regulatory hormones (e.g. insulin, leptin). In the fasted state, and at hourly intervals in each postprandial period, an additional 5 mL will also be drawn to quantify plasma concentrations of hormones implicated in appetite regulation (e.g. ghrelin, peptide YY). Once obtained, this additional 5 mL will be immediately transferred to an EDTA tube to which 50 μL of a 1.0 mM p-hydroxymercuribenzoic (PHMB) acid solution has been added. This will then also be centrifuged before 600 μL of the supernatant is removed and transferred to a sterile tube containing 60 μL of 1 M hydrochloric acid and centrifuged once again. The PHMB and acidified plasma samples will then be aliquoted into two separate 1.5 mL tubes and stored at −80 °C for later analysis. Following the draw of each blood sample, the cannula will be flushed with a 0.9% sodium chloride solution to keep it patent. All samples will be centrifuged (Biofuge Primo R, Heraeus, Germany) at 4 °C for 10 minutes at 3466×*g*.

### Adipose tissue biopsy

Adipose tissue biopsies will be obtained from the subcutaneous abdominal region. For this, a site 5 cm to the left or right of the umbilicus will be sterilised and anaesthetised (1% lidocaine hydrochloride without adrenaline, Hameln Pharmaceuticals, UK) before a sample is obtained using the needle aspiration method [51]. This sample will be cleaned on 100 nm gauze with a sterile 0.9% sodium chloride solution before being separated into aliquots of approximately 250 mg. One aliquot will be immediately disrupted in TRIzol (Invitrogen) using a hand-held homogeniser (Fisher Scientific, CA, USA) and frozen at −80 °C for subsequent analysis. The second aliquot will be immediately frozen at −80 °C. Biopsies obtained in LAB 2 and LAB 3 will be taken from opposing sides of the umbilicus.

### Urine collection

Urine samples will be collected at the outset of each lab session to ensure participants are similarly hydrated for repeated body mass measurements. The samples will be analysed on the day for osmolality, via the freeze-point depression method (Micro Osmometer 3300, Advanced Instruments, MA, USA) and specific gravity (SUR-NE Clinical, Atago, Tokyo). Furthermore, during the 3-hour postprandial period which follows the breakfast meals, total urine output will be collected into containers prepared with 5 mL of a 10% thymol–isopropanol solution. At the end of the 3-hour period, the total volume of urine will be measured with a 1 mL sample extracted and frozen at −80 °C for subsequent analysis of urine urea concentration, as an indicator of protein excretion, to calculate the contribution of protein oxidation to whole body energy metabolism.

### Appetite

Appetite will be measured using visual analogue scales [52] assessing hunger, stomach fullness, desire to eat, thirst and food preference (sweet, salty, savoury, fatty), and two computer-based tasks. For each of the visual analogue scales there will be a question (e.g. how hungry do you feel?) followed by a 100 mm visual scale with the extremes defined (e.g. not at all – as hungry as I have ever felt). Participants shall be asked to place a vertical line on the scale to denote their perception relative to the two extremes, with the distance from the zero point to the marked point measured and recorded by the same researcher for all scales.

The computer-based tasks are a two alternative forced choice task (2AFCT) and an ideal portion size task (IPST) [53]. The 2AFCT comprises a set of 18 foods which are individually photographed in an approximately standard portion on a white plate, or in a plain glass bowl placed on the white plate. In terms of nutrient composition and taste, there are six triplets of foods. The foods within each triplet have a very similar energy density and weight, and across the triplets energy density varies from 1.1 kcal/g to 5.0 kcal/g. Each triplet contains one sweet high-carbohydrate food, one non-sweet high-carbohydrate food and one non-sweet low-carbohydrate food. An example triplet is vanilla ice cream, Singapore noodles and stir-fry sauce, and tuna and mayonnaise. In the task, each food is paired side-by-side once with all of the other foods to give a total of 153 trials (the side on which the foods appears is randomised across trials). For each pair of foods, the participant indicates by depressing the left or right arrow-key on the computer keyboard which food they would ‘choose to eat right now’. Outcome measures are (1) proportion of trials on which a high-sweetness, high-carbohydrate food is chosen, (2) proportion of trials on which a low-sweetness, high-carbohydrate food is chosen, (3) proportion of trials on which a low-carbohydrate food is chosen, and (4) average energy content of the chosen foods for each of these categories. There is a ‘base rate’ for each category because, on some occasions, there will be a choice between two low-carbohydrate foods, for example, but these will differ in energy content/density. For the IPST [54], there are three test foods, vanilla ice cream, Singapore noodles and stir-fry sauce, and tuna and mayonnaise, which are photographed in portions ranging from 20 to 1000 kcal in 20 kcal increments (50 pictures in total). In each of three trials one of these foods is displayed in the middle of the computer screen. Depressing the left arrow-key causes the portion size to decrease (a smaller portion is displayed) whilst pressing the right arrow-key causes the converse. The pictures are loaded with sufficient speed that continuous depression of the left or right arrow key gives the appearance that the change in portion size is animated. Each trial starts with a different and randomly selected portion size. Participants are instructed to ‘Imagine you are offered this food right now. Use the left and right arrow keys to adjust the portion to show how much of it you would eat.’

These measures are designed to complement measures of hormones implicated in appetite control and account for any changes in how satiety signals are interpreted. Furthermore, the two computer tasks will determine whether the different interventions influence preference for sweet or non-sweet carbohydrates, or both. The visual analogue scales shall be completed in the fasted state and at 30 minute intervals following the breakfast meal in LAB 2 and LAB 3. The computer-based tasks will be completed in a fasted state and 45, 105 and 165 minutes after the breakfast meal.

### Data management

Upon providing informed consent, participants will be assigned with a unique identifier that will be used on all data collection documentation to preserve confidentiality. Data recorded in physical form will be digitised and stored in a central file that can only be accessed by the research team. Once a complete dataset has been compiled, manuscripts will be drafted and submitted for publication in appropriate peer-reviewed journals. The criteria that warrant an authorship in resulting publications will be addressed once data analysis has been completed. Once the results have been formally published the trial dataset will be released in anonymised form and an engagement event will be arranged to inform participants of the findings. Should any data or biological samples collected in the present study be needed for ancillary studies, appropriate consent will be sought from participants before proceeding.

A full risk assessment has been conducted by the research team and was subsequently approved by the research sponsor and the NHS Research Ethics Committee (Frenchay). Prior to enrolling, all participants will be fully informed of the potential risks associated with engaging in this study. In particular this will centre upon the symptoms they may experience as a result of prolonged fasting and dietary restriction (e.g. headaches, tiredness, hunger, irritability, spells of dizziness/faintness), as well as the risk of discomfort and infection that accompanies tissue biopsy and blood sampling procedures. Participants will be free to withdraw from the study at any time during their involvement. As neither participants nor researchers are blinded to trial allocation the research team will take responsibility for noting and reporting adverse events to the study sponsor and the ethics committee. Overall conduct of the trial, including adverse event reporting, data management protocols and researcher conduct, will be subject to biannual progress reports by the research sponsor. However, there is no intention to end the trial prematurely based in interim results which, together with the wider data monitoring framework, renders a data monitoring committee unnecessary. Any modifications to the study protocol will be submitted to the trial sponsor and NHS research ethics committee (Frenchay); if approved, publications and trial registrations will be updated accordingly.

### Data analysis

As outlined in the aims of the present study, the primary outcomes will explore the effects of the intervention on postprandial glucose/insulin responses (pre–post) and energy balance – reflected by changes in resting metabolic rate (pre–post), physical activity energy expenditure (monitoring–intervention) and postprandial thermogenesis (pre–post). Secondary outcomes will explore changes from pre- to post-intervention in body composition, substrate oxidation, lipid profile, leptin concentration, c-reactive protein concentration, adipose tissue gene expression, postprandial lipaemia and postprandial appetite regulation/perception. In addition, differences in physical activity intensity and dietary composition between the monitoring and intervention phases will be examined. As it is not the objective of this study to compare these differences between the cohorts, two sets of analyses will be conducted on a per protocol basis -– one for the lean cohort and one for the overweight/obese cohort. Both will centre upon a two-way mixed model analysis of variance featuring a factor for diet allocation and a factor for time-point. This will permit an examination of the effect of each diet on the outcomes of interest (i.e. pre vs. post/monitoring vs. intervention) and whether this effect varied between the three dietary conditions. Pre-existing differences between groups at baseline will be examined using a one-way analysis of variance featuring a factor for diet allocation. Significant differences emerging from these tests will be explored using appropriate post-hoc tests to adjust for multiple comparisons and isolate the source(s) of variance. In addition to these analyses at the group level, individual responses will also be closely scrutinised to examine whether outliers could be affecting the interpretation. Further analysis, such as sub-group analysis, may also be conducted in light of patterns emerging in the final dataset. Baseline characteristics of participants who withdraw during the intervention will also be compared against the final population using *t* tests. Statistical significance will be accepted at *P* < 0.05.

## Discussion

Despite the growing popularity of IMF [55], research exploring its effects on human metabolism remains scarce. The novel examination of postprandial responses and the components of energy balance being employed in this study will highlight the extent to which IMF regimes can impact human metabolism. The isocaloric contrast of IMF and daily calorie restriction will also add a further dimension by thoroughly benchmarking IMF against current nutritional strategies for improving metabolic health. Furthermore, the inclusion of a eucaloric IMF arm will examine whether any observed changes can be attributed to extended fasting alone as opposed to chronic energy imbalance. Finally, in exploring all of these facets in both lean and overweight/obese groups, the present study will also clarify the effect of baseline adiposity on observed changes, which may unravel the discrepancies seen in prior studies [56, 57]. Taken together, this approach will help to provide clearer and more informed public health messages on the safety and efficacy of such diets relative to conventional approaches.

## Trial status

This trial was retrospectively registered on July 6, 2015, at clinicaltrials.gov under identifier NCT02498002 (version: IMF-02). Recruitment commenced on June 8, 2015, and is currently ongoing. It is anticipated that recruitment will be completed by July 2018.

## Additional files

Additional file 1: Participant information sheet. The participant information sheet given to participants following an expression of interest in volunteering. (PDF 528 kb)

Additional file 2: Informed consent form. The informed consent form signed by participants to signify their entry into the study. (PDF 294 kb)

Additional file 3: SPIRIT checklist. (DOC 120 kb)

## Abbreviations

2AFCT: two alternative forced choice task
BMI: body mass index
DEXA: dual-energy x-ray absorptiometry
FMI: fat mass index
IMF: intermittent fasting
IPST: ideal portion size task
PAL: physical activity level
PHMB: p-hydroxymercuribenzoic acid
