# Supplementary material for: Intermittent fasting, energy balance and associated health outcomes in adults: study protocol for a randomised controlled trial
Source: Trials. 2018 Feb 2;19:86. doi: 10.1186/s13063-018-2451-8 (PMC5797418; doi:10.1186/s13063-018-2451-8)
Supplement: Supplementary file 1 — Participant information sheet. The participant information sheet given to participants following an expression of interest in volunteering. (PDF 528 kb) [file 13063_2018_2451_MOESM1_ESM.pdf]

University of Bath
Department for Health

Participant Information Sheet

Project: Impacts of intermittent fasting on energy balance and associated health outcomes

Location: University of Bath, Claverton Down, Bath, BA2 7AY

Investigator: Iain Templeman (i.s.templeman@bath.ac.uk)

Project Background

Obesity is a global health problem which has been linked with conditions like heart disease and diabetes. These conditions are usually treated by reducing the amount of food and drink consumed each day, but this is often unsuccessful because it requires high levels of motivation.

Intermittent fasting has been suggested as another way to tackle these problems, where days of normal eating are spread between days of fasting. You may have heard of the 5:2 diet which is based around this idea. However, despite the growing popularity of intermittent fasting, it is not clear whether it is just another way to lose weight or whether it actually changes how our bodies work.

Our main goal is to look at how intermittent fasting affects the biochemical processes which lead to obesity, including those that affect the storage of fat, the breakdown of sugar, and our appetites. We then plan to compare these changes against those seen in effective diets to see which is better.

Overview

Enrolment
- Read information sheet and ask questions
- Check eligibility and provide informed consent

Monitoring
- 75 minute lab visit
- 4 x 3-day monitoring periods over one month
- Monitor physical activity, body weight and diet.

Lab 1
- Lab visit from 07:30 - 15:00
- Measure body composition and metabolic responses to food
- Assign to diet

Diet
- Complete 20 days of dieting
- Monitor physical activity, body weight and diet.

Lab 2
- Lab visit from 07:30 - 15:00
- Measure body composition and metabolic responses to food
- Complete diet feedback questionnaire

Experimental Protocol

Enrolment

If you are interested in volunteering having read this information sheet and discussed any questions you have with us, we will start by checking that you are eligible for the study. This involves measuring your height and weight, which we can help with, and completing questionnaires on your eating habits, capacity to exercise and general health. If you are eligible then we will guide you through the paperwork to formally enrol in the study.

Monitoring Phase

The first part of the protocol will be a 4 week monitoring phase. This is to make sure your weight is stable and to look at your diet and physical activity levels during your normal routine. While this phase will last for 4 weeks, we will only collect data over 4 x 3-day periods (12 days in total).

This phase will start with a 75 minute visit to the University to collect some starting values. We will ask you to provide a small urine sample to check that you are hydrated before we measure your height, weight and waist circumference. We will also take a blood sample from your arm to check the levels of sugar and fat in your blood.

Lastly, we will need to individually calibrate your monitoring equipment to make sure it is accurate. This will involve being on a treadmill while wearing a mouthpiece as we gradually increase the speed from walking (9 minutes), to uphill walking (6 minutes) to running (4.5 minutes). The idea is see how much energy you use at different exercise intensities, but you can stop the test at any time.

Before leaving, you will be given two monitoring tools. The first will be an 'Actiheart^{TM}' which is an advanced form of heart rate monitor that is attached to the chest to see how much energy you burn through physical activity. The last will be a log book and a compact set of scales to create a diet record so we can calculate your energy and nutrient intake. We will also provide you with a set of bathroom scales to make sure your body weight is stable.

Pre-Intervention Laboratory Protocol (Lab 1)

One month later you will need to come to the University for almost a full day following an overnight fast. You will need to wear light clothing and arrive at 07:30 in a fasted state, for which travel arrangements can be made if needed. We will begin by asking for a small urine sample to check you are hydrated before we measure your height, weight and waist circumference. Following this we will take you to a private lab where you will be asked to recline on an adjustable bed for the rest of your visit. You will have 20 minutes to settle in before we collect expired air samples using a mouthpiece to measure your metabolic rate (how much energy you burn at rest).

At 08:20 a cannula, which is a thin plastic tube, will then be inserted into a vein on your forearm or hand using a small needle for blood sampling throughout your visit. Blood samples will then be taken from the cannula to measure resting levels of sugar, fat and appetite hormones in your blood. We will then move on to take an adipose (fat) tissue biopsy. **This is an optional test which we will discuss with you when you enrol in the study.** If included, an area on the side of your tummy just above the waistband will be sterilised and injected with an anaesthetic (Lidocaine Hydrochloride) to numb the area. Using a needle we can then take a 1 gram sample of adipose tissue from just below the skin without causing any pain. There will be a puncture mark and perhaps bruising, but if left to heal there should be no scarring. Those who choose not to include this will simply continue resting.

Lastly, visual analogue scales, which ask you to rate certain feelings on a scale, will be used to measure perceived hunger and appetite.

At 09:00 you will be given a test breakfast (porridge made with milk and sugar) to be eaten within 10 minutes. Blood samples and visual analogue scales will then be collected at 15, 30, 45, 60, 90, 120 and 180 minutes. Expired air samples will also be collected at 60, 120 and 180 minutes. Throughout the morning we will also collect your urine to see how your body is using different nutrients to provide energy, for which a container with preserving chemicals will be provided.

At 12:30, we will then complete a mixed meal tolerance test, which is used in healthcare to diagnose a range of conditions. We will ask you to place your arm in a padded box which will be heated to 60°C. At 12:30 a blood sample will be taken from the cannula after which you will be asked to drink a small milkshake. We will then draw a 7 ml blood sample every 15 minutes for two hours, after which the cannula will be removed at approximately 14:30.

To conclude this visit we will perform a Dual-Energy X-ray Absorptiometry (DEXA) scan which will involve you lying still for 7 minutes. This is a type of x-ray which will tell us how your weight is split between fat and muscle. While this does use radiation, the risk associated with it is the same as spending a morning in Cornwall. Please be aware that all invasive tests (e.g. fitting cannulas, adipose tissue biopsies) will only be performed by qualified and experienced staff members, although they will not be medical doctors.

Diet Phase

Before leaving the lab you will be randomly assigned to one of three diets, listed below. All three diets will last for 20 days in total, with each 24 hour diet period starting at 15:00. The idea behind this is to make sure that you will always have at least one main meal per day, if not more. In addition, when fasting you are still allowed water and black tea/coffee (no milk or sugar), so if you struggle without caffeine in the morning there is no need to worry.

Daily calorie restriction	Reduce normal energy intake by 25% every day.
Intermittent fasting with weight loss	Alternate between 24 hour periods of fasting and feeding with 150% of normal energy intake on fed days.
Intermittent fasting without weight loss	Alternate between 24 hour periods of fasting and feeding with 200% of normal energy intake on fed days.

For the first and last weeks of your diet we will ask you to wear an ‘Actiheart^{TM}’ monitor to see whether the diet affects the amount of physical activity you do whilst also noting any changes to the content of your normal diet in a new log book. Having finished the last day of your diet at 15:00 you will need to prepare for your post-intervention laboratory visit by returning to your normal diet for one day.

Post-Intervention Laboratory Protocol (Lab 2)

As before you will need to arrive at 07:30 in a fasted state for which travel arrangements can be made if needed. We will also ask you to wear the same clothing as before while we will repeat the protocol described earlier for your pre-intervention visit. Comparing the results from these two visits will allow us to see how the diet has affected your body.

Feedback

Four weeks after this post-intervention visit we will arrange a feedback session with you to discuss all available results, including energy intake, physical activity, body composition and metabolic rate. Blood results from the meal tests will follow at a later stage. Where convenient you will be asked to provide a weight measurement as well to see how sustainable any weight changes might be.

Summary

When written out in full this study will seem very intense because there are a lot of measurements described in a lot of detail. However, please remember that this will all be spread across 7-8 weeks. In addition, the diets have been designed based on feedback from an earlier study to make them easier to follow. This has been done by making sure that you get at least one main meal per day and allowing you to drink caffeine when fasting.

This is a great opportunity to get involved with an interesting and highly talked-about research area. We hope that the detailed measurements being used will not only help you to better understand your lifestyle and physiology, but that they will help us understand the advantages and disadvantages of intermittent fasting.

So whether you have an interest in nutrition or just want to take a detailed look at your lifestyle and health, please don't hesitate to contact us.

Monitoring Equipment

An important part of this project is understanding how the different diets affect our bodies as we follow them. To help with this we are using an 'Actiheart™' which is an advanced form of heart rate monitor that is attached to the left side of your chest using two adhesive pads (figure 1). The device will initially be fitted and calibrated by a member of the research team, but you will occasionally need to adjust the device and change the adhesive pads yourself. The device simply clips on to the adhesive pads allowing you to easily detach and reattach it as needed, but to remove it completely you simply peel off the pads. This needs to be worn for 4 x 3-day periods during the monitoring phase and for the first and last weeks of your diet phase.

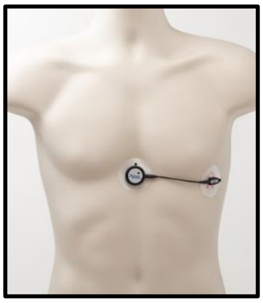

Figure 1: Actiheart™ monitor

At certain points during the study we will also need to gather information on your diet, for which you will be given a log book and some compact digital scales. This will allow you to tell us the type and quantity of food and drink you consume, from which we can give you an accurate breakdown of your energy and nutrient intake.

A researcher will be contactable by phone at all times during the study if you have any issues.

Potential for Inconvenience

The main inconvenience of this study is the need to diet as all three diets will involve restriction. Volunteers have found that the diets become easier as they get used to them, but the first few days may be challenging for some. You should also think about the duration of the study, as it is a 7-8 week commitment. However, there will only be a maximum of 20 hours where you will need to visit the University and we can be flexible on dates. Finally, keeping a record of your food and fluid intake can also be inconvenient, but a full record is only needed for a total of 12 days and a digital record can be used if you find this easier.

If you find participating too difficult or inconvenient once you have started you will be free to withdraw from the study at any time.

Potential Risks

Although there are no specific side effects of fasting, you may get symptoms similar to those you get after skipping a meal. Examples include tiredness, irritability, headaches, dizziness and nausea. In the event of minor side effects, you will be asked contact the research team, but if more severe problems occur then a small amount of a sugary food should be eaten first.

In terms of the laboratory protocols, intravenous cannulation can cause bruising/redness which may take a few days to fade. Less common risks include an infection or an embolism, where an obstruction becomes lodged within the vessel and partially blocks the blood flow. Adipose tissue biopsies can also present a risk of infection as once again the skin is punctured with a needle. It is also common to get minor bleeding, bruising or swelling following an adipose tissue biopsy (figure 3) which can take over a week to fade. **This is why this test is optional.** We will check for unusual responses after the biopsy, but you will be able to contact us if you have concerns after your visit. To minimise the risk associated with these procedures, they will only be performed by experienced staff.

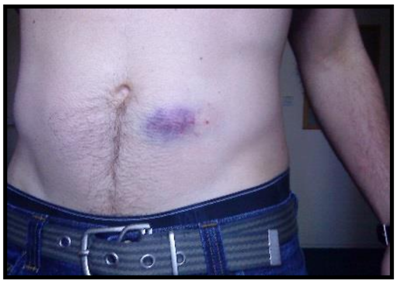

Figure 3: Example of bruising after an adipose tissue biopsy.

Another risk relates to the volume of blood to be sampled during the study, approximately 320 ml. This is just over half the amount taken during a blood donation so it is unlikely that you will get any side effects from this, but low blood pressure, dizziness, tiredness and headaches are possible.

In addition, the Dual Energy X-ray Absorptiometry scan used to measure your body composition does give a small dose of radiation. The dose given is similar to spending a morning in Cornwall, which has an estimated 1 in 10 million risk of causing harm. As two scans will be performed as part of the study the risk is 2 in 10 million, which is lower than the risk for a normal diagnostic X-ray.

Some participants may also experience irritation from the adhesive pads used to attach the 'Actiheart™' to the chest. However, this can be avoided by changing the pads every 2 days and cleaning the area beneath. Alternatively a non-adhesive chest strap can be used where necessary. Lastly, as information which can be used to identify you is being recorded alongside details about you, there is a small risk to confidentiality. To minimise this we will give you a unique identification code which will be used on all data collection documents instead of your name. Therefore, only the researchers will be able to link your data directly to you.

Potential Benefits

Unfortunately we cannot pay you for participating in this study, but there are still benefits to getting involved. Once your data is processed **you will be fully informed of your personal results**, including:

- Basal Metabolic Rate – How many calories your body burns at rest
- Activity Energy Expenditure – How many calories you burn through physical activity
- Body Composition – How many kilograms of muscle and fat you have in your body
- Energy Intake – How many calories you eat/drink per day
- Nutrient Intake – How many grams of key nutrients you consume per day

- Appetite Regulation – How your body regulates your appetite before and after meals
- Postprandial Responses – How your body handles the nutrients you eat (i.e. sugar, fat)

Where possible your results will be presented alongside the normal ranges, with each measurement **explained in straight-forward terms**. As part of this we can also use the data from your diet records and physical activity profiles to provide **personalised feedback on your diet and exercise habits**. This feedback could then be used to create **guidelines for improving your lifestyle and health**. We will also give you the results of all three groups and the whole study as they become available. In the event that any unusual results are identified you will be advised to follow this up with your GP.

Finally, volunteering for this project will give you the opportunity to get involved in **an exciting field of research**. Intermittent fasting is a topical but largely un-researched area, and given the thorough measurements being used there is a **chance that new findings will be made which could be used to develop public health policies**.

On behalf of the research team I would like to thank you for your interest in our study. If you would like to talk about the study further, ask questions or volunteer please do not hesitate to contact us using the details below:

Mr Iain Templeman
Human Physiology Research Group
1 West 3.102
University of Bath
Claverton Down
Bath, BA2 7AY

Email: i.s.templeman@bath.ac.uk
