# Supplementary material for: Intermittent fasting, energy balance and associated health outcomes in adults: study protocol for a randomised controlled trial
Source: Trials. 2018 Feb 2;19:86. doi: 10.1186/s13063-018-2451-8 (PMC5797418; doi:10.1186/s13063-018-2451-8)
Supplement: Supplementary file 2 — Informed consent form. The informed consent form signed by participants to signify their entry into the study. (PDF 294 kb) [file 13063_2018_2451_MOESM2_ESM.pdf]

University of Bath
Department for Health

Informed Consent Form

Project: Impacts of intermittent fasting on energy balance and associated health outcomes

Location: University of Bath, Claverton Down, Bath, BA2 7AY

Investigator: Iain Templeman (i.s.templeman@bath.ac.uk)

Participant Name: ___________________________________

Gender: __________________

Date of Birth: __________________

Contact Email: ___________________________________

Contact Number: ___________________________________

Please initial each statement that is appropriate (if you require more information before consenting to participate or are unsure on any of the points below, please discuss this with the researcher before proceeding)**:**

☐ I confirm that I have read and understood the accompanying 'Participant Information Sheet' dated 01/04/2015 (version 1.4) for the above study.

☐ I confirm that a member of the research group has discussed all aspects of the study with me with at a level of detail and a vocabulary that is appropriate.

☐ I confirm that I have been given the opportunity to ask questions relating to the study and all of its elements and have received satisfactory answers.

☐ I am aware that no therapeutic effects (e.g. weight loss) are guaranteed by my participation in this study.

☐ I understand that my participation is voluntary and that I am free to withdraw at any point without having to give a reason, or compromising my legal rights.

☐ I give consent for my samples to be appropriately stored and used until all necessary analysis for the above study is completed.

☐ I understand that my data from the study may need to be looked at by individuals from the University of Bath, regulatory authorities, or those from NHS Trusts where it is relevant to my participation in the research. I give permission for these individuals to access my records where appropriate.

☐ I agree to take part in the study stated above.

Participant Preferences
This section of the form relates to optional elements of the study which will have to be requested at this point in order to be included. However, please note that should you choose to withdraw from these elements of the study at any point without withdrawing from the study as a whole, your decision will be accepted by the researchers without justification or consequences.

Please initial all appropriate statements (if you require more information before consenting to participate or are unsure on any of the points below, please discuss this with the researcher before proceeding):

☐ I confirm that I have completed the 'Entry Criteria Questionnaire' (version 1.4, 01/04/2015) and do not suffer from any of the listed contraindications for the administration of Lidocaine Hydrochloride.

☐ I confirm that I have been fully informed of the procedure for an 'Adipose Tissue Biopsy' and the risks that accompany it.

☐ I am aware that the 'Adipose Tissue Biopsy' is an optional procedure that is not required in order for me to participate in this study

☐ I agree to provide a maximum of two 'Adipose Tissue Biopsies' during my involvement in the above study

Participant Declaration
I fully understand what is involved in taking part in this study. Any questions I have about the study, or my participation in it, have been answered to my satisfaction. I have been informed that I am free to withdraw my consent and discontinue participation at any time. If I decide to withdraw I understand that it will not have any undesirable consequences. I have had my attention drawn to the following guidelines for research involving human subjects (an electronic copy of which is available upon request):

Council for International Organizations of Medical Sciences & the World Health Organization. (2002). *International Ethical Guidelines for Biomedical Research Involving Human Subjects.* Geneva: CIOMS.

It has been made clear to me that, should I feel that these regulations are being infringed or that my interests are otherwise being ignored, neglected, or denied, I should inform the Director of Postgraduate Studies within the Department for Health at the University of Bath who will undertake to investigate my complaint.

Participant Signature: ___________________________ Date: ______________

Print Name: __________________________________

Researcher Signature: __________________________ Date: ______________

Print Name: __________________________________
